# Familial aggregation and heritability: a nationwide family-based study of idiopathic inflammatory myopathies

**DOI:** 10.1136/annrheumdis-2021-219914

**Published:** 2021-06-15

**Authors:** Weng Ian Che, Helga Westerlind, Ingrid E Lundberg, Karin Hellgren, Ralf Kuja-Halkola, Marie Holmqvist

**Affiliations:** 1 Clinical Epidemiology Division, Department of Medicine, Solna, Karolinska Institutet, Stockholm, Sweden; 2 Division of Rheumatology, Department of Medicine, Solna, Karolinska Institutet, Stockholm, Sweden; 3 Rheumatology, Gastro. Derm, Rheuma, Karolinska Institutet Hospital, Stockholm, Sweden; 4 Department of Medical Epidemiology and Biostatistics, Karolinska Institutet, Stockholm, Sweden

**Keywords:** dermatomyositis, polymyositis, epidemiology

## Abstract

**Objectives:**

The magnitude of the genetic contribution to idiopathic inflammatory myopathies (IIMs) is unknown. In this project, we aimed to investigate the familial aggregation and heritability of IIM.

**Methods:**

This is a family-based study using nationwide healthcare register data in Sweden. We matched each patient with IIM to individuals without IIM, identified their first-degree relatives and determined the IIM status among all first-degree relatives. We estimated the adjusted ORs (aORs) of familial aggregation of IIM using conditional logistic regression. In addition, we used tetrachoric correlation to estimate the heritability of IIM.

**Results:**

We included 7615 first-degree relatives of 1620 patients with IIM diagnosed between 1997 and 2016 and 37 309 first-degree relatives of 7797 individuals without IIM. Compared with individuals without IIM, patients with IIM were more likely to have ≥1 first-degree relative affected by IIM (aOR=4.32, 95% CI 2.00 to 9.34). Furthermore, the aOR of familial aggregation of IIM in full siblings was 2.53 (95% CI 1.62 to 3.96). The heritability of IIM was 22% (95% CI 12% to 31%) among any first-degree relatives and 24% (95% CI 12% to 37%) among full siblings.

**Conclusions:**

IIM has a familial component with a risk of aggregation among first-degree relatives and a heritability of about 20%. This information is of importance for future aetiological studies and in clinical counselling.

**Key messages**

**What is already known about this subject?**

- Previous family-based studies and genome-wide association studies of idiopathic inflammatory myopathies (IIMs) suggest a genetic predisposition to IIM. However, how much genetics contribute to IIM remains unclear.

**What does this study add?**

- Family history of IIM in first-degree relatives is associated with the occurrence of IIM.
- Additive genetic factors explain 22% of the variation of IIM in the Swedish population within the study period. Compared with the SNP-based heritability of IIM, our heritability estimate of IIM is much higher, implying that there are unknown genetic variants associated with IIM to be discovered.

**How might this impact on clinical practice or future developments?**

- Our findings provide new insight into the genetic predisposition to IIM and implicate the potential of using family history of IIM as an indicator in the diagnostic workup.

## Introduction

Idiopathic inflammatory myopathies (IIMs) are rare systemic inflammatory diseases of partly unknown pathogenesis.^1^ Onset and progression of IIM is influenced both by environmental and genetic factors, but the exact interplay between these factors remains unclear.^1 2^ Recently, genome-wide association studies (GWASs) confirmed that alleles in the human leucocyte antigen (HLA) 8.1 ancestral haplotype—HLA-DRB1*03:01 and HLA-B*08:01—are important genetic risk factors for IIM.^3–6^ Other genes outside the HLA region such as PTPN22 have also been suggested. Although there are established genetic risk factors for IIM, the impact of genetics on the risk of developing IIM is unknown.

Investigating the degree to which a disease aggregates in families (familial aggregation) and how much of the phenotypic variance of a disease is explained by the genetic variance in a population (heritability) may provide insight on the genetic contribution to that disease.^7 8^ Evidence in the literature indicating familial aggregation of IIM is conflicting. There are several case reports and one population-based family study supporting familial aggregation,^9–16^ while other family-based studies failed to detect any.^17–19^

Previously published heritability estimates in IIM are based on GWAS data, where about 8.3% of the phenotypic variance for polymyositis (PM) and 5.5% of the variance for dermatomyositis (DM) is explained by the genetic variance in the studied population.^20^ These estimates are unlikely to reflect the true heritability of IIM, partly since they are based on data from GWAS where only single nucleotide polymorphisms (SNPs) of selected loci are genotyped, but they can serve as a lower bound.^21 22^ Heritability can also be estimated using family data.^8^ Estimates from this method are generally considered as the upper bound of heritability since they may include the influence of other similarities, besides genetics, among relatives. As far as we know, there are no studies reporting the heritability of IIM using family-based designs.

Knowing more about the genetic predisposition to IIM could improve our understanding of the underlying aetiology of the disease. We therefore set out to investigate the familial aggregation and heritability of IIM using nationwide register data in Sweden.

## Methods

### Study setting and data sources

The healthcare system in Sweden is primarily tax funded, which ensures equal access for all residents. The *National Patient Register* (*NPR*) was established in 1964 and prospectively collects data on inpatient care with virtually 100% coverage since 1987. Since 2001, it includes around 80% of all non-primary outpatient visits, and missing visits are primarily from private practice.^23^ Patients with IIM are exclusively managed by hospital-based rheumatologists.

The *Multi-Generation Register* (*MGR*) includes information on parents, siblings and children of all individuals born later than 1931 and registered in Sweden since 1 January 1961. The ascertainment of parents of individuals born in Sweden in 1952 and afterwards is above 90%.^24^

The *Total Population Register* (*TPR*), founded in 1968, has data on nearly 100% of births and deaths in Sweden and is often used to randomly sample comparators from the general population in research.^25^

### Study population

We included all patients having ≥1 hospitalisation with IIM as main diagnosis between 1997 and 2000 in the NPR. Between 2001 and 2016, when both inpatient and outpatient data were available, we included all patients having ≥2 outpatient visits or hospitalisations with IIM whereof at least one had IIM as the main diagnosis. We only considered International Classification of Diseases (ICD), 10 codes M33 and G72.4 from internal medicine, rheumatology, dermatology, neurology or paediatrics department. Patients are only assigned these ICD codes when the medical assessments are completed, and the diagnosis of IIM is certain. This includes excluding potential IIM-mimics.^26^ The algorithm used to identify IIM has been found to be robust, and the ICD codes have been validated with a positive predictive value up to 96% using clinician-entered diagnosis from the Swedish Rheumatology Quality Register as gold standard.^27 28^ We further categorised IIM into DM (M33.1), other IIM (M33.2, M33.9 and G72.4) and juvenile IIM (JIIM) (M33 or G72.4 with age ≤18 years at diagnosis). We randomly matched each patient with IIM with up to five individuals without IIM from the TPR. The matching factors were sex, birth year and residential area at the time of IIM diagnosis in their matched patient with IIM. All individuals without IIM were alive and living in Sweden at match date. We only included patients with IIM and individuals without IIM (collectively referred to as index individuals) who were born in Sweden in 1932 and onwards to increase ascertainment of their biological first-degree relatives. The study population mainly includes individuals of Caucasian ethnicity. Via linkage to the MGR and the TPR, we ascertained their parents, full siblings and offspring, as well as data on sex and birth year of first-degree relatives. As exposure status to IIM could not be determined before 1987, we only included first-degree relatives who were alive in 1987. Lastly, we excluded index individuals without any first-degree relatives identified.

### Identification of IIMs in first-degree relatives

In the primary analyses, we required first-degree relatives to have ≥1 visit with IIM as main diagnosis in the NPR between 1987 and 2017 to be considered as exposed. The ICD 9 codes used for the period between 1987 and 1996 were 710D and 710E. We also performed a sensitivity analysis where we used a stricter definition, the same definition of IIM as we used for index individuals, to determine IIM in first-degree relatives.

### Identification of muscular dystrophies and metabolic myopathies

To examine the risk of misdiagnosing inherited myopathies as IIM, we went through all the main diagnoses of inpatient and outpatient visits registered between 1987 and 2017 of individuals in the family units affected by IIM to identify diagnoses of muscular dystrophies and metabolic myopathies (online supplemental table 1).

10.1136/annrheumdis-2021-219914.supp1 Supplementary data

### Statistical methods

We described the demographic characteristics and the family structure between patients with IIM and individuals without IIM. Variables were described using mean (SD) and median (first and third quartiles) or frequencies with proportions.

#### Familial aggregation of IIMs

In this family-based design, we treated IIM in index individuals as the outcome and IIM in first-degree relatives as the exposure. We used logistic regression conditioning on the matching cluster to estimate the adjusted ORs (aORs) of having first-degree relatives affected by IIM in patients with IIM compared with individuals without IIM. We performed the analyses by number of affected relatives and by treating each first-degree kinship pair as an independent unit and using a robust sandwich variance estimator to control SEs for familial clustering. We additionally adjusted for sex and birth year of first-degree relatives in the second modelling method.

#### Heritability of IIMs

Given the population data used in this study, we estimated the heritability of IIM overall and among full siblings using the tetrachoric correlation based on several assumptions: (1) a normally distributed liability model of IIM with a disease threshold; (2) no assortative mating; (3) that the genetic variance was solely due to additive genetic effects; and (4) that the only similarity between first-degree relatives was genetics. For example, no environmental factors shared between siblings were assumed to contribute to disease liability.^7 29^ Thus, the provided heritability estimate is a narrow sense heritability where only additive genetic effects, the sum of average effects of disease-associated alleles, are considered.^7^ We created a 2×2 contingency table presenting the concordant and discordant relative pairs of patients with IIM and individuals without IIM. Since the ascertainment of individuals without IIM was a subsample of the entire population, we adjusted the calculation by the observed prevalence of IIM in Sweden (0.014%)^27^ and used the observed OR of IIM associated with having first-degree relatives affected by IIM for calculation. These calculations have been described in detail elsewhere.^30^ The intraclass correlation coefficient divided by the degree of relatedness, 0.5 for first-degree relatives, was considered the heritability of IIM.^7^ We repeated the analyses by varying the prevalence between 0.004% and 0.024% to test the robustness of heritability to changes of IIM prevalence in the population.

#### Sensitivity analyses

We repeated all analyses using the above-mentioned stricter definitions to define IIM in first-degree relatives. This was done to test the robustness of the main analyses. As the outpatient register was not nationwide until 2001, we repeated the analyses by including only first-degree relatives who were alive in 2001.

We primarily used SAS V.9.4 (SAS Institutet) for data management and analyses, except for tetrachoric correlations, which were estimated in R V.3.6.1^31^ using the R-package *polycor*.^32^ As this was an entirely register-based study, we did not collect patient consent.

### Patient and public involvement

We did not involve patients in any stage of this study.

## Results

**Figure 1:**
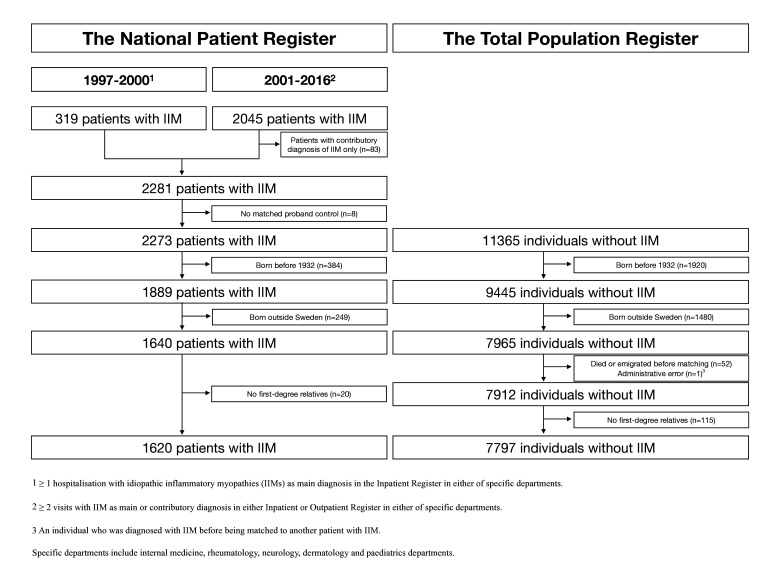
Identification of patients with idiopathic inflammatory myopathies (IIMs) and individuals without IIM from the National Patient Register and the Total Population Register, respectively.

This study included 1620 patients with IIM and 7797 individuals without IIM (figure 1 and online supplemental table 2). Of 1620 patients with IIM, 951 (59%) were women, and the median birth year was 1949. The median age at inclusion was 57 years. Eight per cent of the patients were diagnosed with JIIM, 31% with DM and 61% with other IIM (table 1). The number of patients with prevalent IIM ascertained by calendar year since 2001 were generally stable (online supplemental figure 1). Other characteristics and family structure were similar between the patients with IIM and individuals without IIM (table 2). The mean (SD) number of first-degree relative per family unit was 4.7 (2.2) in patients with IIM and 4.8 (2.1) in individuals without IIM.

**Table 1:** Characteristics of patients with idiopathic inflammatory myopathies (IIMs) and individuals without IIM*

	Patients with IIM (n=1620)	Individuals without IIM (n=7797)
Women, n (%)	951 (59)	4639 (60)
Birth year, median (Q1–Q3)	1949 (1941–1964)	1949 (1941–1964)
Living in Southern Sweden, n (%)	1342 (83)	6455 (83)
Age at inclusion, median (Q1–Q3)	57 (44–66)	57 (44–65)
IIM subtypes, n (%)		
Juvenile IIM†	128 (8)	–
Dermatomyositis‡	501 (31)	–
Other IIM§	991 (61)	–

*Q1: the first quartile; Q3: the third quartile.

†Age at diagnosis ≤18 years of age.

‡With diagnostic code M33.1 and age at diagnosis >18 years of age.

§With diagnostic code M33.2, M33.9 or G72.4 and age at diagnosis >18 years of age.

**Table 2:** Family structures of patients with idiopathic inflammatory myopathies (IIMs) and individuals without IIM, and demographic characteristics of their first-degree relatives*

	Patients with IIM		Individuals without IIM	
Any first-degree relatives, n, mean±SD	7615	4.7±2.2	37 309	4.8±2.1
Parents	2306	1.4±0.8	11 414	1.5±0.7
Women, n (%)	1253 (54)		6314 (55)	
Birth year, median (Q1–Q3)	1926 (1916–1943)		1926 (1916–1943)	
Full siblings	2464	1.5±1.5	11 685	1.5±1.5
Women, n (%)	1238 (50)		5863 (50)	
Birth year, median (Q1–Q3)	1951 (1943–1962)		1950 (1943–1963)	
Offspring	2845	1.8±1.3	14 210	1.8±1.3
Women, n (%)	1335 (47)		6960 (49)	
Birth year, median (Q1–Q3)	1975 (1966–1987)		1975 (1967–1989)	

*Q1: the first quartile; Q3: the third quartile.

### Familial aggregation of IIMs

Thirteen (0.8%, 9 family units) of 1620 patients with IIM had at least one first-degree relative affected by IIM versus 16 (0.2%, 16 family units) in 7797 individuals without IIM, corresponding to an aOR of 4.32 (95% CI 2.00 to 9.34) (table 3). All cases of familial IIM were 45 years of age or above when diagnosed, and none of the individuals had any visits indicating a main diagnosis of muscular dystrophies or metabolic myopathies. Seven of the nine family units affected by IIM had concordant diagnoses of other IIM. The aOR of familial aggregation decreased to 2.61 but remained significant with a narrower 95% CI (1.80 to 3.79) when all first-degree kinship pairs were treated as independent units. Proportions of IIM were higher in all types of first-degree kinship in patients with IIM compared with individuals without IIM. We only estimated the aOR (2.53, 95% CI 1.62 to 3.96) for full siblings since the number of cases of IIM in individuals with affected parents or offspring in both groups were <5.

**Table 3:** Adjusted ORs (aORs) of having first-degree relatives affected by idiopathic inflammatory myopathies (IIMs) in patients with IIM compared with individual without IIM*

	Patients with IIM, n/N (%)	Individuals without IIM, n/N (%)	aOR† (95% CI)	aOR‡ (95% CI)
≥1 relative	13/1620 (0.80)	16/7797 (0.21)	4.32 (2.00 to 9.34)	–
Any first-degree relatives	13/7615 (0.17)	16/37309 (0.04)	2.61 (1.80 to 3.78)	2.61 (1.80 to 3.79)
Parents	2/2306 (0.09)	5/11414 (0.04)	–	–
Full siblings	9/2464 (0.37)	10/11685 (0.09)	2.54 (1.62 to 3.99)	2.53 (1.62 to 3.96)
Offspring	2/2845 (0.07)	1/14210 (0.01)	–	–

*≥1 relative: comparison between patients with IIM and individuals without IIM; any first-degree relatives, parents, full siblings and offspring: comparison between relative pairs of patients with IIM and relative pairs of individuals without IIM.

†Controlled for sex, birth year and residential area of index persons.

‡Controlled for sex, birth year and residential area of index persons, sex and birth year of first-degree relatives.

### Heritability of IIMs

**Figure 2:**
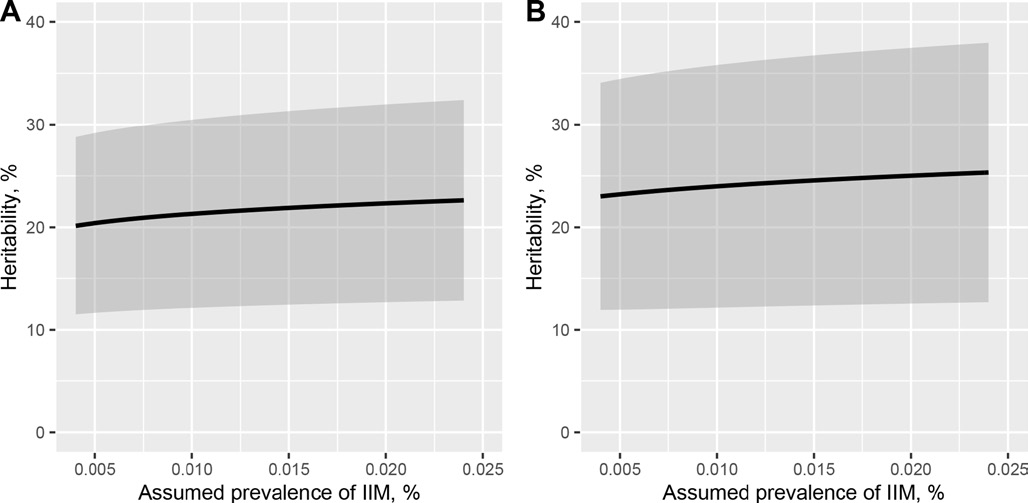
Point estimates with 95% CIs (grey shading) of heritability of IIM using assumed prevalence of IIM ranging from 0.004% to 0.024%, with a fixed interval of 0.0002% for (A) any first-degree relatives and (B) full siblings only. IIM, idiopathic inflammatory myopathy.

The overall heritability of IIM among any first-degree relative was 22% (95% CI 12% to 31%) when the underlying prevalence of IIM was assumed to be 0.014% and the observed OR in the 2×2 table was 3.99. With the same assumed prevalence of IIM, the point estimate of the heritability of IIM among full siblings increased slightly to 24% (95% CI 12% to 37%) with an observed OR of 4.28 in the contingency table. Varying the assumed prevalence of IIM in the population between 0.004% and 0.024% did not affect the result (figure 2).

#### Sensitivity analyses

Results of analyses using a stricter definition to define IIM among first-degree relatives or analyses including only relatives alive in 2001 were in line with the findings of the primary analyses (online supplemental tables 3–6).

## Discussion

This is the first population-based family study that estimates the genetic contribution to IIM based on familial aggregation and heritability. We found that having at least one first-degree relative affected by IIM was strongly associated with the risk of developing IIM, and above one-fifth of the phenotypic variance of IIM could be attributed to additive genetic variance in the Swedish population.

Prior to our study, several studies have attempted to examine the familial aggregation of IIM with family-based^17 18^ and population-based methods.^19^ None of the studies found any significant associations, probably due to insufficient statistical power. The study of Ginn *et al* 17 including 151 first-degree relatives of 21 patients with IIM reported only one familial DM. Similarly, there were only three cases of familial DM in a study including first-degree to third-degree relatives of 304 children with juvenile DM, resulting in a non-significant OR=3.00 with wide 95% CI (0.31 to 28.9).^18^ A Danish nationwide register study including 949 patients with dermato-polymyositis presented a familial OR of 3.9 (95% CI 0.6 to 27.7) for parents or siblings.^19^ In our study, which increased the number of patients with IIM by 70%, we found a similar but statistically significant point estimate of familial aggregation of IIM, suggesting that the non-significances in previous studies might be mainly due to insufficient statistical power. Recently, a study by Thomsen *et al* 16 comprising 2668 patients with an ICD code suggesting IIM between 1964 and 2012 also observed familial aggregation of IIM for parents and siblings (standardised incidence ratio=4.03, 95% CI 1.27 to 8.35), a finding that is in line with our results.

Our heritability estimate of 22% was higher than previously published SNP-based heritability (5.5% for DM and 8.3% for PM).^20^ This is to be expected for several reasons. First, in SNP-based heritability, the SNPs with mild or moderate effects on a trait that do not reach statistical significance are not included in the calculations,^21^ something that could lead to an underestimation of the heritability even though the genome-wide complex trait analysis (GCTA) was used to compensate for this problem.^33 34^ Second, the SNP-based heritability in IIM was estimated based on SNPs presented on the Immunochip, where SNPs are selected based on GWAS findings of 12 autoimmune diseases excluding IIM. The Immunochip therefore is not specific to IIM.^22^ That is, the SNP-based heritability of IIM already published is likely to only represent a portion of the actual heritability of IIM. In addition, Golan *et al* 35 have previously demonstrated that the GCTA method could underestimate the heritability when a disease is rare.

In our present study, we estimated heritability by using correlations among first-degree relatives. When doing so, we made several assumptions including that genetics are the only similarity among first-degree relatives. This assumption is unlikely to be true, and if there are environmental factors contributing to disease within a family, we might overestimate the heritability of IIM. However, the results in our study show that the heritability of IIM in all first-degree relatives among those who in general share less environment growing up (ie, parents and offspring are usually exposed to different childhood environments) was similar to that in full siblings who often do share environment growing up. This supports that our findings are not results where shared environmental factors largely explain the variance of IIM.

Estimates of heritability should be interpreted with caution. The heritability estimated in our study tells us the extent of *variation* of IIM explained by additive genetic *variation* and not of how much genes influence the risk of IIM.^7^ We should therefore not infer that the probability of IIM being passed from parent to offspring is 22%, a concept that can be expressed as inheritability, or that genetics is less important than non-genetic factors in the development of IIM. Though heritability estimates tell little or even nothing about causal effect of genes on a disease, it is still an important starting point to explore the genetic contribution to a disease where today there are no available methods to estimate inheritability of a complex trait.^36^

Our study suggests that IIM generally has a lower risk of familial aggregation and heritability than some other autoimmune diseases. This may suggest that the pathogenesis of IIM is more complex than the pathogenesis of other autoimmune diseases. For example, the overall familial risk of any first-degree relatives and heritability for rheumatoid arthritis in the Swedish population is 3.2 (95% CI 3.0 to 3.3) and 40%, respectively.^30^ For organ-specific autoimmune diseases, even higher estimates have been presented; in a Swedish twin study, the familial risk of coeliac disease was 124 (95% CI 81 to 129) in monozygotic twins.^37^ Given that we found a higher heritability of IIM compared with the previously reported SNP-based heritability, there may still be unknown genes contributing to the pathogenesis of IIM. One direction for future research would be to do whole genome sequencing of patients with IIM to discover novel genetic variants associated with IIM, and hence further improve our understanding of the aetiology of IIM.

Our study has several limitations. Due to lack of serological data, we could not examine the familial aggregation and heritability stratified by antibody profiles, nor could we perform analyses stratified by clinical subtypes, age at onset and sex since there were insufficient cases of familial IIM. We could not control for dependence between observations in the tetrachoric correlations, which could mean that our heritability CIs might be too narrow. This would however not affect the point estimate. Also, the generalisability of our findings to other populations should be taken with caution. Genetic variants of IIM vary somewhat across ethnicities.^38^ For example, HLA-DRB1*12:02, associated with IIM in a Korean population, is rarely found in Caucasian populations.^39^ The heritability estimate is specific to population, time and environment.^36^

Lastly, we could not completely eliminate the risk of misdiagnosing inherited myopathies as IIM. However, we found no diagnoses of muscular dystrophies or metabolic myopathies in the years before and after the IIM diagnosis in individuals with familial IIM in our study. In Sweden, each patient undergoes a muscle biopsy before a diagnosis of IIM is set, and the clinical awareness of IIM mimics among rheumatologists, neurologists and neuropathologists is high.^26^ Thus, even if misclassification of IIM mimics as IIM could not entirely be ruled out, we think the risk is low in our clinical setting.

Despite these limitations, our study provides novel insight of the genetic contribution to IIM with validated data and robust analyses. Using high-quality nationwide register data, we have a representative sample of IIM, and our study is well powered. As our data were prospectively collected in the registers, we avoid weaknesses usually associated with case–control studies, such as recall bias. The matched design resulted in even family structures and distributions of sex and birth year of first-degree relatives between patients with IIM and individuals without IIM, minimising bias due to these factors on the estimates.

Our findings do not only improve our understanding of the genetic contribution to IIM but it may also have important clinical implications. In current diagnostic workup of IIM, information on family history of muscle weakness and autoimmunity is useful.^1 26^ With our results, we add knowledge on how to assess family history in IIM in the diagnostic workup of IIM.

In conclusion, this study suggests that family history of IIM could influence the risk of IIM and that there are additional genetic risk markers to be identified. This information is important for both our aetiological understanding of IIM and clinical counselling.

## Data Availability

No data are available.
