# Supplementary material for: Familial aggregation and heritability: a nationwide family-based study of idiopathic inflammatory myopathies
Source: Ann Rheum Dis. 2021 Jun 15;80(11):1461–6. doi: 10.1136/annrheumdis-2021-219914 (PMC8522465; doi:10.1136/annrheumdis-2021-219914)
Supplement: Supplementary data [file annrheumdis-2021-219914supp001.pdf]

Familial aggregation and heritability - a nationwide family-based study of idiopathic inflammatory myopathies

SUPPLEMENTARY MATERIALS

Che WI, *et al*. *Ann Rheum Dis* 2021; 80:1461–1466. doi: 10.1136/annrheumdis-2021-219914

Supplementary Table 1. The International Classification of Diseases (ICD) codes of myopathies dystrophies and metabolic myopathies

**Diseases**	**ICD 9**	**ICD 10**
Myopathies dystrophies[a]	359B	G71.0
Metabolic myopathies[b]	271A	E74.0

a Includingdysferlinopathies and facioscapuhumeral musculardystrophy.

b Including McArdle disease and acid maltase deficiency.

Supplementary Table 2. The frequencies and proportions of other relevant diseases diagnosed in 83 excluded individuals with only contributory diagnoses of IIM

**Diseases**	N (%)
Any of the diseases below[a]	58 (69.9%)
Systemic inflammatory diseases[b]	48 (57.8%)
Multiple sclerosis	1 (1.2%)
Autoimmune thyroid diseases	13 (15.7%)
Celiac disease	2 (2.4%)
Myasthenia gravis	5 (6%)
Non-inflammatory myopathies[c]	9 (10.8%)

a Including systemic inflammatory diseases, multiple, sclerosis, autoimmune thyroid diseases, celiac disease, myasthenia gravis and non-inflammatory myopathies.

b Including rheumatoid arthritis, systemic systemic erythematosus lupus, systemic sclerosis, Sjögren's syndrome and other systemic connective tissues diseases.

c Including muscular diseases defined with International Classification of Diseases Tenth Revision codes G70-G73.

Che WI, *et al. Ann Rheum Dis* 2021; 80:1461–1466. doi: 10.1136/annrheumdis-2021-219914

Supplementary Table 3. Adjusted odds ratios (aORs) of having first-degree relatives affected by idiopathic inflammatory myopathies (IIM) in patients with IIM compared to individual without IIM, using the strict definition of IIM[a]

	**Patients with IIM, n/N (%)**	**Individuals without IIM, n/N (%)**	**aOR[b] (95% CI)**	**aOR[c] (95% CI)**
≥1 relative	10/1620 (0.62)	14/7797 (0.18)	4.02 (1.70-9.49)	-
Any first-degree relatives	10/7615 (0.13)	14/37309 (0.04)	2.59 (1.67-4.01)	2.59 (1.67-4.01)
Parents	1/2306 (0.04)	5/11414 (0.04)	-	-
Full siblings	8/2464 (0.32)	9/11685 (0.08)	2.47 (1.53-3.99)	2.47 (1.54-3.99)
Offspring	1/2845 (0.04)	0/14210 (0)	-	-

a ≥1 relative: Comparison between patients with IIM and individuals without IIM; Any first-degree relatives, parents, full siblings and offspring: Comparison between relative pairs of patients with IIM and relative pairs of individuals without IIM. Strict definition of IIM: ≥ 1 hospitalisation with IIM as main diagnosis between 1997 and 2000 in the NPR. Between 2001 and 2016, ≥2 visits with IIM as main or contributory diagnosis. Individuals with IIM as contributory diagnoses only were excluded. Only International Classification of Diseases 10 codes M33 and G72.4 from internal medicine, rheumatology, dermatology, neurology or paediatrics department were considered.

b Controlled for sex, birth year and residential area of index persons.

c Controlled for sex, birth year and residential area of index persons, sex and birth year of first-degree relatives.

Che WI, *et al. Ann Rheum Dis* 2021; 80:1461–1466. doi: 10.1136/annrheumdis-2021-219914

Supplementary Table 4. Heritability of idiopathic inflammatory myopathies (IIM), using the strict definition of IIM[a]

	Exposed cases	Unexposed cases	Odds ratio	Prevalence (%)	Tetrachoric correlation	Heritability (CI), %
All first-degree relatives	10	7605	3.50	0.014	0.0967	19 (9-30)
Full siblings	8	2456	4.23	0.014	0.1197	24 (11-37)

a Strict definition of IIM: ≥ 1 hospitalisation with IIM as main diagnosis between 1997 and 2000 in the NPR. Between 2001 and 2016, ≥2 visits with IIM as main or contributory diagnosis. Individuals with IIM as contributory diagnoses only were excluded. Only International Classification of Diseases 10 codes M33 and G72.4 from internal medicine, rheumatology, dermatology, neurology or paediatrics department were considered.

Supplementary Table 5. Adjusted odds ratios (aORs) of having first-degree relatives affected by idiopathic inflammatory myopathies (IIM) in patients with IIM compared to individual without IIM, including only first-degree relatives alive in 2001[a]

	Patients with IIM, n/N (%)	Individuals without IIM, n/N (%)	aOR[b] (95% CI)	aOR[c] (95% CI)
≥1 relative	13/1605 (0.81)	15/7722 (0.19)	4.71 (2.14-10.34)	-
Any first-degree relatives	13/6840 (0.19)	15/33550 (0.04)	2.72 (1.53-4.83)	2.70 (1.86-3.92)
Parents	2/1619 (0.12)	5/8079 (0.06)	-	-
Full siblings	9/2397(0.38)	9/11347 (0.08)	2.74 (1.74-4.34)	2.72 (1.73-4.30)
Offspring	2/2824 (0.07)	1/14124 (0.01)	-	-

a ≥1 relative: Comparison between patients with IIM and individuals without IIM; Any first-degree relatives, parents, full siblings and offspring: Comparison between relative pairs of patients with IIM and relative pairs of individuals without IIM.
b Controlled for sex, birth year and residential area of index persons.
c Controlled for sex, birth year and residential area of index persons, sex and birth year of first-degree relatives.

Che WI, *et al. Ann Rheum Dis* 2021; 80:1461–1466. doi: 10.1136/annrheumdis-2021-219914

Supplementary Table 6. Heritability of idiopathic inflammatory myopathies (IIM), including only first-degree relatives who were alive in 2001

	Exposed cases	Unexposed cases	Odds ratio	Prevalence (%)	Tetrachoric correlation	Heritability (95% CI), %
All first-degree relatives	13	6827	4.26	0.014	0.1150	23 (13-33)
Full siblings	9	2388	4.75	0.014	0.1310	26 (14-38)

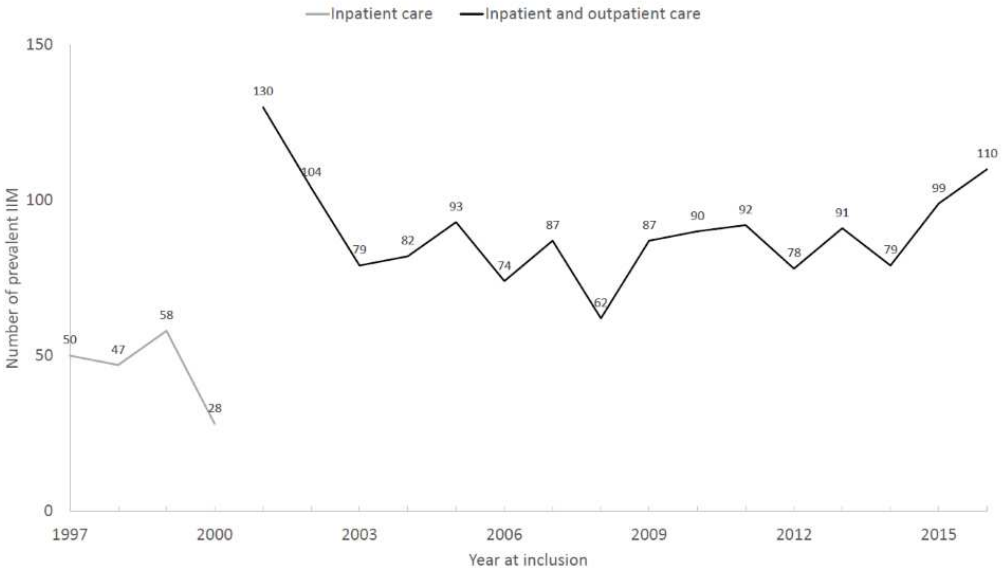

Supplementary Figure 1. The trends of ascertainment of prevalent IIM by calendar year. *Grey line:* only data in inpatient care was available between 1997 and 2000; *black line:* data on inpatient and outpatient care were available between 2001 and 2016.

Che WI, *et al. Ann Rheum Dis* 2021; 80:1461–1466. doi: 10.1136/annrheumdis-2021-219914
